# Dissemination of Metallo-β-Lactamase-Producing *Pseudomonas aeruginosa* in Serbian Hospital Settings: Expansion of ST235 and ST654 Clones

**DOI:** 10.3390/ijms24021519

**Published:** 2023-01-12

**Authors:** Jovana Kabic, Gianuario Fortunato, Ivone Vaz-Moreira, Dusan Kekic, Milos Jovicevic, Jovan Pesovic, Lazar Ranin, Natasa Opavski, Célia M. Manaia, Ina Gajic

**Affiliations:** 1 Institute of Microbiology and Immunology, Faculty of Medicine, University of Belgrade, Dr Subotica Starijeg 1, 11000 Belgrade, Serbia; 2 CBQF—Centro de Biotecnologia e Química Fina—Laboratório Associado, Universidade Católica Portuguesa, Escola Superior de Biotecnologia, Rua Diogo Botelho 1327, 4169-005 Porto, Portugal; 3 Faculty of Biology, University of Belgrade, Studentski trg 16, 11000 Belgrade, Serbia

**Keywords:** high-risk clones, carbapenem-resistant *Pseudomonas aeruginosa*, *bla_NDM_*, *bla_PER_*, *bla_GES_*, MLST, WGS

## Abstract

This nationwide study aimed to investigate the molecular characteristics of metallo-β-lactamase (MBL)-producing *Pseudomonas aeruginosa* in Serbia, underlying resistance mechanisms, the genetic context of detected MBL genes, and the clonal relationship between isolates harboring genes-encoding MBL. Overall, 320/5334 isolates collected from 2018 to 2021 were identified as *P. aeruginosa*. Carbapenem-resistant *P. aeruginosa* (CRPA) were screened for the presence of *bla_VIM_*, *bla_IMP_*, and *bla_NDM_*, genes whereas MBL-positive isolates were tested for the presence of the *bla_CTX-M-2_*, *bla_PER_*, *bla_TEM_*, *bla_SHV_*, *bla_VEB_*, and *bla_GES_*. Multilocus sequence typing and phylogenomic analysis were performed for *P. aeruginosa*-producing MBL. The majority of the *P. aeruginosa isolates* were recovered from the lower respiratory tract (n = 120; 37.5%) and wound specimens (n = 108; 33.75%). CRPA isolates accounted for 43.1% (n = 138) of the tested isolates, 31 out of them being *bla_NDM-1_*-positive (22.5%). The colistin resistance rate was 0.3%. MLST analysis revealed the occurrence of ST235 (n = 25) and ST654 (n = 6), mostly confined to Serbia. The distribution of beta-lactamase-encoding genes in these isolates suggested clonal dissemination and possible recombination: ST235/*bla_NDM-1_*, ST235/*bla_NDM-1_*/*bla_PER-1_*, ST654/*bla_NDM-1_*, ST654/*bla_NDM-1_*/*bla_PER-1_*, and ST654/*bla_NDM-1_*/*bla_GES-5_*. High-risk clones ST235 and ST654 identified for the first time in Serbia, are important vectors of acquired MBL and ESBL and their associated multidrug resistance phenotypes represent a cause for considerable concern.

## 1. Introduction

*Pseudomonas aeruginosa* is an important opportunistic human pathogen involved in a variety of community and hospital-acquired infections, ranging from respiratory tract infections, endocarditis, and urinary tract infections to septicemia [1]. *P. aeruginosa* has been recognized as a common cause of ventilator-associated pneumonia, particularly in intensive care units (ICU) [2]. High adaptability, genetic plasticity, and versatile and ubiquitous features make *P. aeruginosa* a very concerning and difficult-to-treat pathogen. In addition, the treatment of *P. aeruginosa* infections has become a great challenge due to its intrinsic resistance to many antimicrobial agents and the ability to easily acquire antibiotic resistance through mutational changes or the acquisition of resistance genes via horizontal gene transfer [3]. *P. aeruginosa* is one of the six leading mortality-causing pathogens, which together were responsible for 929,000 deaths attributable to antimicrobial resistance (AMR) and 3.57 million deaths associated with AMR in 2019 globally [4]. Thus, multidrug-resistant *P. aeruginosa* (MDR-PA) and extensively drug-resistant *P. aeruginosa* (XDR-PA) strains are becoming major clinical threats worldwide due to their association with high mortality, long hospital stays, and increased costs [5]. The World Health Organization (WHO) has listed carbapenem-resistant *P. aeruginosa* (CRPA) as one of the three bacterial species in which there is a critical need for the development of new antibiotics to treat infections [6]. Likewise, *P. aeruginosa* is included in the group ESKAPE, an acronym introduced to designate a group of bacteria that escape the action of antibiotics: *Enterococcus faecium*, *Staphylococcus aureus*, *Klebsiella pneumoniae*, *Acinetobacter baumannii*, *P. aeruginosa,* and *Enterobacter* species [7]. Data from the annual report of Central Asian and European Surveillance of Antimicrobial Resistance (CAESAR) showed that CRPA isolates accounted for 55% of invasive *P. aeruginosa* in Serbia in 2020 [8]. Carbapenem resistance in *P. aeruginosa* has shown to be multifactorial, including (i) acquisition of carbapenemase-encoding genes through horizontal gene transfer [9], (ii) deficiency or repression of the porin (OprD) for carbapenems [10], (iii) overexpression of *mexAB-oprM* efflux pump [11], and (iv) overexpression of the chromosomal gene (*ampC*) encoding the *P. aeruginosa* intrinsic cephalosporinase [12]. However, the acquired carbapenemase-encoding genes need special attention because horizontal gene transfer through mobile genetic elements, such as transposons, plasmids, and integrative and conjugative elements, could accelerate the dissemination of the CRPA. Indeed, acquired extended-spectrum β-lactamases (ESBLs) located on mobile genetic elements, such as serine-β-lactamases of molecular classes A and D and metallo-β-lactamases (MBLs) of class B, are important resistance mechanisms in *P. aeruginosa* [13]. Considering the role of *P. aeruginosa* in the development of nosocomial infections, the analysis of the genetic context of the MBL genes is essential in identifying associations with other genes conferring antimicrobial resistance and revealing potential routes of dissemination. The first report on the *P. aeruginosa* producing MBL in Serbia and the Balkan region was the detection of isolates carrying *bla_NDM-1_* gene in 2011 [14].

Epidemiological studies based on multilocus sequence typing (MLST) may help to understand and identify circulating MDR *P. aeruginosa* strains worldwide. Previously published reports showed that *P. aeruginosa* sequence type (ST) 235 is the predominant global clinical lineage [15]. Besides ST235, most of the carbapenemase-producing *P. aeruginosa* reported worldwide belong to ubiquitous high-risk clones, such as ST111, ST175, and ST654 [16,17]. However, in Serbia, there is a lack of data regarding circulating STs and the genomic epidemiology of CRPA isolates.

To address the prevalence and underlying mechanisms of antibiotic resistance and the epidemiology of *P. aeruginosa*, the objectives of the current study were (i) to determine the antimicrobial resistance profile of *P. aeruginosa* isolated from hospitalized patients across Serbia, (ii) to investigate the prevalence of *P. aeruginosa* producing MBLs, (iii) to analyze the genetic context of detected MBL genes and (iv) to investigate the clonal relationship between isolates harboring genes encoding MBL.

## 2. Results

Out of 5334 collected bacterial isolates, 320 (6%) corresponded to *P. aeruginosa*. The isolates were recovered from hospitalized patients with an average age of 59.72 years within the age range of 2 months and 88 years old. The clinical samples included lower respiratory tract samples (n = 120; 37.5%), wound specimens (n = 108; 33.75%), urine (n = 71; 22.2%), and blood (n = 21; 6.6%). Most of the *P. aeruginosa* isolates were obtained from the ICU (n = 178; 55.6%). Detailed information concerning specimen types, patient characteristics, and hospital units is listed in Table 1.

### 2.1. Antimicrobial Resistance

**Figure 1:**
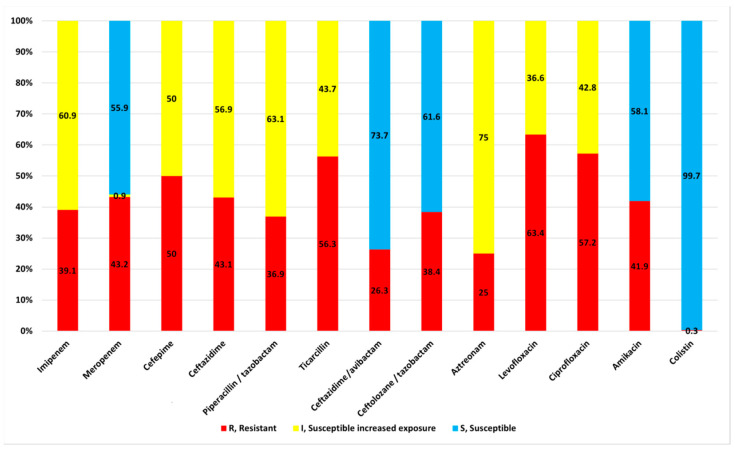
Antimicrobial resistance of the 320 clinical isolates of *Pseudomonas aeruginosa*.

The highest resistance prevalence (>50%) was observed for fluoroquinolones, levofloxacin, and ciprofloxacin. Resistance prevalence against the tested beta-lactam antibiotics ranged from 25–56.3%, being 43.1% for carbapenems (Figure 1). The lowest resistance rates were observed for ceftazidime—avibactam, aztreonam, and colistin. Indeed, 99.7% of the isolates displayed in vitro susceptibility to colistin. All isolates had MIC values of colistin < 2 mg/L, except one isolate that had a MIC value > 16 mg/L.

Overall, 154 (48.1%) and 114 (35.6%) out of 320 *P. aeruginosa* isolates were MDR and XDR, respectively. The most common MDR phenotype observed was non-susceptibility to ceftazidime, piperacillin-tazobactam, ticarcillin, meropenem, levofloxacin, and ciprofloxacin (42%).

Among the 320 isolates of *P. aeruginosa*, 138 (43.1%) were CRPA, being resistant to meropenem and/or imipenem. The respective MICs were >8 mg/L for meropenem and/or >4 mg/L for imipenem. Of note, 23 out of 138 (16.7%) isolates of CRPA were susceptible to amikacin.

The frequency of CRPA isolates recovered from hospital wards (n = 138) was as follows: ICUs—n = 57 (41.3%), cardiovascular surgery—n = 32 (23.2%), general surgery—n = 10 (7.2%), neurosurgery—5 (3.6%), orthopedic surgery—n = 4 (2.9%), plastic surgery—n = 2 (1.4%), internal medicine—n = 10 (7.2%%), geriatric department—n = 9 (6.5%), department for COVID-19-positive patients—n = 6 (4.3%), and department of oncology—n = 3 (2.2%).

The 138 CRPA isolates were identified as adequate candidates for the detection of the MBL-encoding genes.

### 2.2. Molecular Detection of MBL- and ESBL-Encoding Genes

Among the 138 CRPA isolates, 31 (22.5%) harbored MBL-encoding genes, namely *bla_NDM-1_* (Supplementary Figure S1). *P. aeruginosa* harboring *bla_NDM_* were obtained from four hospitals located in three cities (Supplementary Figure S2). The highest number of MBL genes was identified in isolates from patients in ICUs (n = 12; 38.7%), departments for cardiovascular surgery (n = 9; 29.7%), or corona care centers (n = 5; 16.1%). The *bla_VIM_* and *bla_IMP_* genes were not identified in any of the tested isolates.

**Figure 2:**
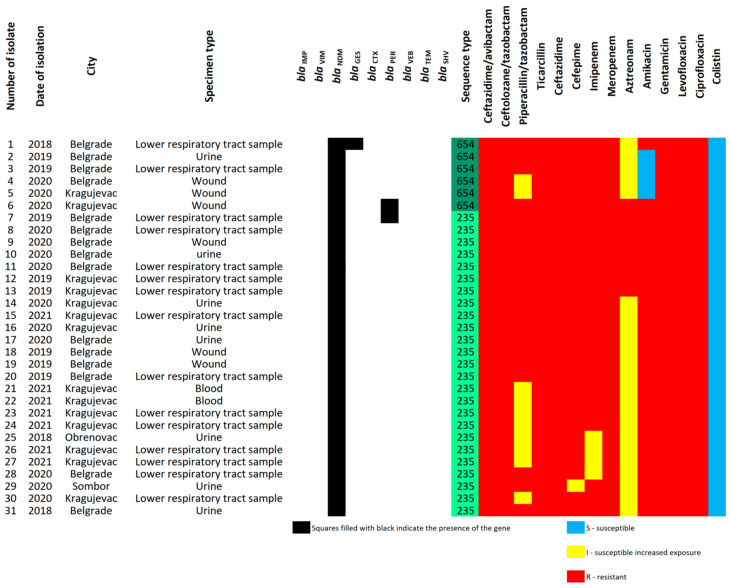
Presence/absence matrix of antimicrobial resistance genes and corresponding sequence types (STs) among 31 *Pseudomonas aeruginosa* isolates harboring *bla_NDM_* gene.

Only three of the 31 isolates (9.7%) found to be *bla_NDM_*-positive carried ESBL-encoding genes. Two isolates harbored simultaneously *bla_NDM-1_* and *bla_PER-1_*, whereas one isolate carried *bla_NDM-1_* and *bla_GES-5._* Among the tested CRPA isolates, *bla_CTX-M_*, *bla_TEM_*, *bla_SHV_*, and *bla_VEB_* genes were not detected (Figure 2).

### 2.3. Multilocus Sequence Typing Analysis

**Figure 3:**
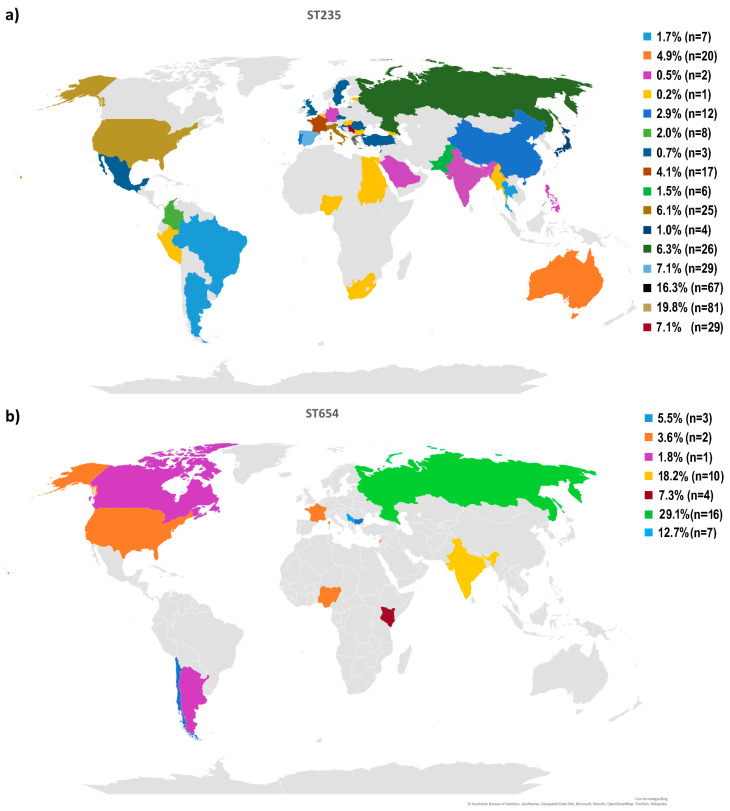
Geographical distribution of the *Pseudomonas aeruginosa* ST235 (**a**) and ST654 (**b**) high-risk clones based on the data available in the Pseudomonas Genome Database (Date accessed 2 January 2023). Colors represent the percentage of clones ST235 and ST654 among isolates of *P. aeruginosa* within a country, as indicated in the geographic map.

MLST analysis was performed for all *bla*_NDM-1_ positive *P. aeruginosa* isolates (n = 31). Among them, 25 and 6 isolates were classified as ST235 and ST654, respectively (Figure 3). One isolate exhibiting resistance to all antimicrobial agents tested in the study, including colistin, belonged to ST235. In addition, two isolates co-harboring *bla_PER-1_* and *bla_NDM-1_* were assigned to ST235 (n = 1) and ST654 (n = 1), whereas the isolate harboring *bla_GES-5_* gene was ST654 (n = 1). In summary, CRPA types identified in this study in Serbia were the following: ST235/*bla_NDM-1_*, ST235/*bla_NDM-1_/bla_PER-1_*, ST654/*bla_NDM-1_*, ST654/*bla_NDM-1_/bla_PER-1_*, and ST654/*bla_NDM-1_/bla*_GES-5_. Genotypes ST235/*bla_NDM-1_* and ST654/*bla_NDM-1_* were found in at least three different cities across Serbia, indicating dissemination throughout the country.

The geographic distribution of genomes and the antibiotic resistance profiles of genomes affiliated with ST235 and ST654 were assessed based on the genomes available in the Pseudomonas Genome Database (Figure 3). From the public database were retrieved all available sequences and information of ST235 and ST654 *P. aeruginosa* isolates. The *P. aeruginosa* ST235 (n = 384) isolates presented a worldwide distribution, mostly in the United States of America (USA) (21.1% of the analyzed isolates). Serbia represented 7.1% of the ST235 isolates available in the online database (Figure 3a). Although submitted in the lower number in the online database (n = 49), most of the ST654 isolates came from Russia (29.1%), followed by India and Serbia (18.2% and 12.7%) (Figure 3b). Analyzing the antibiotic resistance genes profile in both ST235 and ST654, isolates were annotated with different aminoglycoside resistance genes and carbapenemase resistance genes of different classes. Moreover, *bla_OXA-396_* was observed in ST654 isolates, while the *bla_OXA-488_* gene was present in all genomes belonging to ST235 (Supplementary Figure S3).

The ST235 strains’ antibiotic resistance profile indicates the ARGs *bla_GES_* (31 isolates), *bla_NDM_* (30 isolates), and *bla_VIM_* (29 isolates) as the main carbapenem resistance genes harbored. Instead, ST654 isolates presented as the main carbapenems resistance gene *bla_NDM_* (10 isolates) followed by *bla_GES_* (five isolates) and *bla_VIM_* (two isolates).

### 2.4. Phylogenomic Analysis of bla_NDM_-Positive Strains

Details on genome sequencing and assembly quality of four randomly selected *bla_NDM_*-positive *P. aeruginosa* (NDM1_1, NDM1_2, NDM1_3, and NDM1_4) isolates can be found on the National Center for Biotechnology Information site, under BioProject Accession number PRJNA753000. Briefly, the genome sizes ranged from 6.9 to 7.1 Mbp, organized in 1–35 contigs, and coverages ranging from 100–300 x, as shown in Supplementary Table S2. No plasmids were detected among the four *bla_NDM_*-positive *P. aeruginosa* isolates.

**Figure 4:**
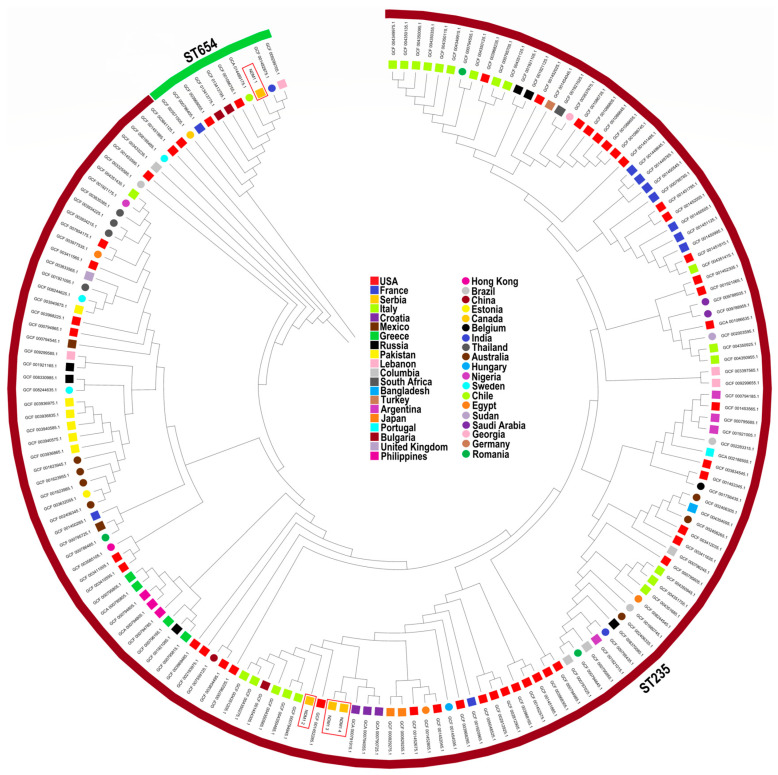
Phylogenetic tree constructed by calling SNPs from core gene alignment of the 165 high-risk clones of *Pseudomonas aeruginosa* (ST235 and ST654) using the neighbor-joining method with 1000 bootstraps. The different countries can be distinguished by different shapes and color codes displayed in the figure legend; the fragments with different colors in the outer ring represent corresponding sequence types (STs), as shown.

The results of phylogenomic analysis of the four genomes of *P. aeruginosa* harboring the *bla_NDM_* gene together with 161 previously published genomes of the same STs with a known geographical location, available in the NCBI Pathogen Detection database (Supplementary Table S3) are illustrated in Figure 4.

Overall, the phylogenetic tree of the 165 *P. aeruginosa* isolates could be divided into two clades according to STs, as illustrated in Figure 4. The ST235 isolates NDM1_2, NDM1_3, and NDM1_4 grouped were closely related to isolates from Croatia, Italy, the USA, and Bulgaria, with detected SNP differences ranging from 59 to 1157 (Supplementary Table S4). The ST654 isolate NDM1_1 was grouped with isolates from Lebanon, India, Chile, and the USA, with detected SNP differences ranging from 447 to 4048 (Supplementary Table S4).

### 2.5. The Genetic Context of the Detected MBL Genes

**Figure 5:**
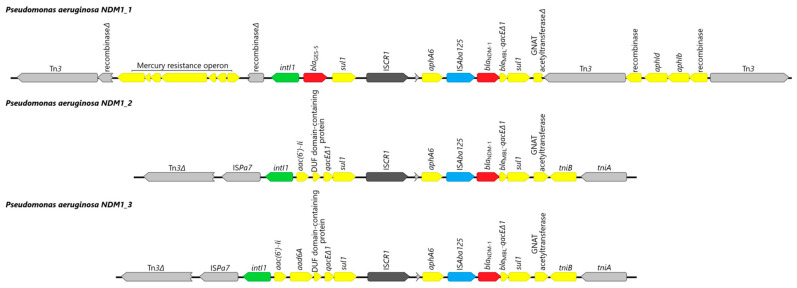
Schematic representation of the *bla*_NDM-1_ region in three analyzed *Pseudomonas aeruginosa* isolates. Δ, an interrupted gene; genes and their transcription orientations are indicated by arrows.

The genetic contexts of the three sequenced *P. aeruginosa* harboring *bla_NDM-1_* gene have been analyzed and shown in Figure 5.

Because the *bla_NDM_* gene in the NDM1_4 isolate was located at the very start of the assembled contig, its genetic context was not examined.

The *bla*_NDM-1_ gene of NDM1_1, NDM1_2, and NDM1_3 is contained within a class 1 integron-bearing insertion sequence (IS) common region 1 (IS*CR1*) (Figure 5). IS*CR1* element is followed by the partial transposase gene IS*Aba14* (accession no. JQ080305.1), the aminoglycoside resistance gene *aphA6*, the transposase IS*Aba125* gene, the *bla*_NDM-1_ gene, the truncated bleomycin/*qac* resistance gene, and the *sul1* end of a class 1 integron.

In NDM1_2 and NDM1_3 isolates, the region upstream of IS*CR1* corresponds to a class 1 integron, with the *intI1* gene followed by several gene cassettes: the genes *aac(6′)Ii* and *aadA6* encoding the aminoglycoside-modifying enzymes AAC-6′(I) and AADA6, respectively, and DUF domain-containing protein. This last cassette is followed by a truncated *qac*/*sul* conserved sequence. Upstream of this complex class 1 integron, IS*Pa7* transposase was detected, as well as incomplete transposase belonging to the Tn3 family.

In the NDM1_1 isolate, the region upstream of IS*CR1* also corresponds to a class 1 integron, with the *intI1* gene, but is followed by a *bla*_GES-5_ gene and gene cassette *sul1.* Upstream of class 1 integron, the mercury resistance operon was detected between partial recombinase genes. In the region downstream of IS*CR1*, aminoglycoside resistance genes *aph(6*′)*Id* and *aph(6′)Ib* were identified, located between two Tn3 family transposases.

Of note, in all four genomes, the aminoglycoside resistance gene *aphA6*, encoding enzyme aminoglycoside 3′-phosphotransferase type VI (Aph(3′)-VI) conferring resistance to amikacin, was detected. In three analyzed genomes, it was located upstream of IS*Aba125* and *bla_NDM-1_* gene. Other aminoglycoside resistance genes identified in four sequenced isolates include *aadA6* (n = 3), *aph(3′)-IIb* (n = 3), *aph(6′)Id* (n = 1), *aph(6′)Ib* (n = 1).

## 3. Discussion

This study presents the first Serbian nationwide multicenter study, providing data on the molecular epidemiology of CRPA isolates recovered from different hospitals throughout the country. Moreover, we assessed the global phylogenetic analysis of the *P. aeruginosa* ST235 and ST654 using four genomes of strains analyzed in the current study together with those available in online genome databases.

In the current study, two-thirds of the isolated *P. aeruginosa* were from wounds and the lower respiratory tract. Due to its ability to grow in simple aqueous solutions, *P. aeruginosa* easily contaminates respiratory medical devices, anesthesia equipment, intravenous fluid, and even distilled water [18]. In the current study, ICU stay seems to be a significant risk factor for infection with MDR *P. aeruginosa* due to the use of invasive devices, such as mechanical ventilation and central lines. Such a finding is in accordance with the previously published report [19].

Alarmingly, in the present study, 43% of the tested isolates were CRPA. Similar results were reported by Radovanovic et al. who found that the resistance rate of hospital isolates of *P. aeruginosa* in Serbia during 2017/2018 was 41.7% [20]. Moreover, a higher resistance rate of 69.5% was assessed by the European Centre for Disease Prevention and Control in 2020 [21]. Based on the previously published reports, we can speculate that the overuse of carbapenems has led to the emergence of carbapenem resistance [22]. Despite aminoglycosides remaining useful antipseudomonal agents, resistance to these drugs continues to be a therapeutic concern, as these genes may be located on mobile genetic elements and thus can be transferred horizontally [23]. Genomic analysis of the present study revealed the simultaneous presence of the aminoglycoside resistance gene *aphA6*, conferring resistance to amikacin, upstream of *bla_NDM_* gene in the same isolates, suggesting dissemination of resistance to aminoglycosides and carbapenems in the hospital environment. An interesting observation in this study was that only 61 out of 138 (44.2%) of the CRPA isolates were resistant to aztreonam. This might be due to the fact that aztreonam is not a substrate for class B β-lactamases [24]. In addition, the retained aztreonam activity in some MDR *P. aeruginosa* despite resistance to other antipseudomonal β-lactams may be explained by the absence of class A ESBL in those isolates. Aztreonam is the only available monobactam that has intermediate activity against *P. aeruginosa* and can be a useful alternative to aminoglycosides or third-generation cephalosporin in patients with serious Gram-negative infections. The resistance rates of the tested *P. aeruginosa* to ceftazidime–avibactam and ceftolozane-tazobactam were comparable to those observed in Hungary in 2017, 33.6 and 32.4, respectively) [25]. According to the results obtained in this study, colistin is considered the best choice for the treatment of MBL-producing CRPA, since the overall assessed resistance rate was less than 1%. The obtained result is much lower than colistin resistance found in Qatar (3.4%) or Egypt (21.3%) [26,27]. Although known for its toxicity, colistin is being reintroduced to the treatment protocols, and the current international consensus is that the optimal use of this drug would be the option for the treatment of infections with MDR Gram-negative bacilli, particularly for *P. aeruginosa* and *A. baumannii* [28].

The *bla_NDM-1_* was the only MBL gene detected in the present study. It was found in 9.7% of the 320 isolates of *P. aeruginosa*. Remarkably, the first report of NDM-positive *P. aeruginosa* was detected in Serbia [14]. However, in most countries, the most frequently reported MBLs globally are VIM and IMP types, whereas NDM has been detected only occasionally [29,30].

In the present study, ESBL-encoding genes detected were *bla_PER-1_* and *bla*_GES-5_*,* found in 6.5% and 3.2% of the tested *bla_NDM_*-positive isolates, respectively. It is noteworthy that the PER-1-type ESBLs are the first reported ESBLs in *P. aeruginosa* and like most other ESBLs can hydrolyze different types of beta-lactam antibiotics except for carbapenem and cephamycin [31]. To the best of our knowledge, the first occurrence of *bla_PER-1_*-positive infections in patients from Serbia was reported in 2008 among a small cluster of patients (n = 4) admitted to one hospital in Belgrade [32]. The identification of PER-1 producers in several European countries, such as Italy and Turkey, also in the Far East, suggests their proceeding dissemination [33,34,35,36]. Globally, GES-type-producing *P. aeruginosa* have already been identified [37]. However, this is the first study that identified *bla_GES-5_*-producing *P. aeruginosa* in Serbia.

The results of our study cast a new light on the genetic characteristics of the *P. aeruginosa* harboring beta-lactamases in Serbia. They all belonged to the dominant clone ST235 and the less prevalent ST654. Overall, these findings are in accordance with findings reporting the global emergence of the widespread *P. aeruginosa* ST235 clone [15,38,39]. Curiously, an NDM-1-producing *P. aeruginosa* ST235 strain was isolated in France from the urine culture of a patient hospitalized in Serbia 3 months earlier (2012) and in Italy from a patient with sepsis who had been hospitalized previously in Serbia (2013) [40,41]. Of note, the *bla_NDM-1_* gene in ST235 isolates NDM1_2 and NDM1_3 was detected in the same genetic environment as previously described in *P. aeruginosa* isolates from France and Italy [40,41]. To the best of our knowledge, this is the first detection of the *P. aeruginosa* strain ST654/*bla_NDM-1_* and ST654/*bla_NDM-1_/bla_GES-5_* in Serbia. According to the genomic data that are currently available on ST654, it seems that it is mostly confined to Russia, India, and Serbia, possibly representing the hotspots for this clone. Similarly, strain ST654, co-harboring *bla_NDM-1,_* and *bla_GES-5_* was also isolated in Bulgaria [42], and the *bla_NDM_* gene was also located in a class I integron In1884.

A few limitations of this study should be mentioned. First, the lack of comprehensive clinical data, which could be used to make a difference between colonization and infection. Second, it was impossible to distinguish nosocomial from community-acquired infections, although the genetic relatedness of the isolates indicates that the source of the infection might be the hospital environment. When interpreting the data, these limitations should be considered, and further studies are warranted to clarify these issues.

## 4. Materials and Methods

### 4.1. Bacterial Isolates

**Figure 6:**
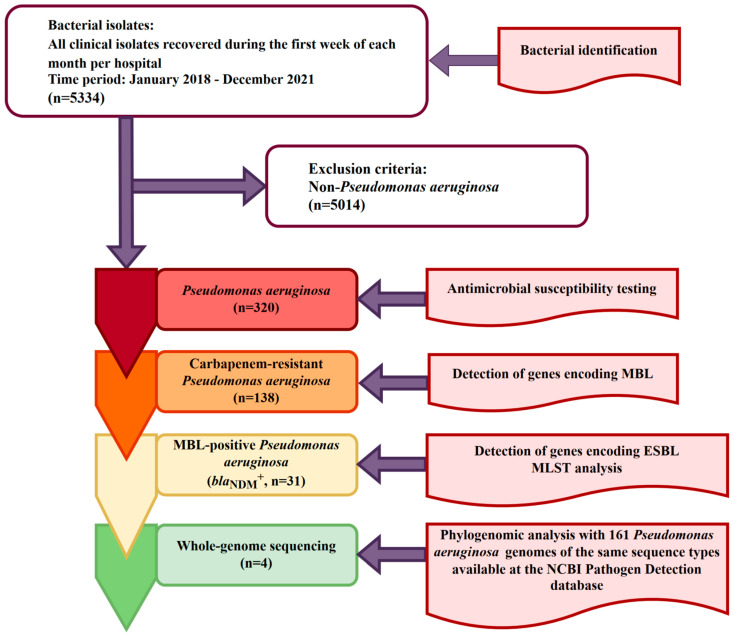
Flowchart of the process of bacterial isolates selection for phenotypic and genotypic characterization; inclusion criteria was the isolation of the *Pseudomonas aeruginosa* from non-redundant clinical samples (one per infected patient) obtained during the routine laboratory work. MBL—Metallo-β-lactamase; ESBL—Extended-spectrum beta-lactamase; MLST—Multilocus sequence typing.

A total of 320 non-redundant isolates of *P. aeruginosa* obtained from patients with bacterial infections, admitted to 11 hospitals throughout Serbia and recovered during the first week of each month from 2018 to 2021, were included in the study. The ethical committee of the Medical Faculty, University of Belgrade (1550/IX-16) approved the study. A flowchart showing inclusion and exclusion criteria for the selection of bacterial isolates is shown in Figure 6.

The bacteria were isolated from various clinical samples using appropriate differential and selective media for *Pseudomonas* and classical microbiological culturing techniques. *P. aeruginosa* were identified by the VITEK2 system (bioMérieux, Marcy-l’Étoile, France), analytical profile index procedure (API 20NE test; bioMérieux, Brussels, Belgium), and MALDI-TOF (Matrix assisted laser desorption-ionisation time of flight) mass spectrometry, according to the manufacturer’s instructions. Pure bacterial cultures identified as *P. aeruginosa* (n = 320) were shipped on Amies transport medium to the coordinating laboratory at the Institute of Microbiology and Immunology of the Medical Faculty in Belgrade for further phenotypic and molecular testing. All isolates were stored at −70 °C in skim milk broth (Merck, Germany) until used in subsequent experiments.

### 4.2. Antibiotic Susceptibility Testing

Antimicrobial susceptibility was tested by employing disk diffusion, gradient test, and broth microdilution test according to the European Committee on Antimicrobial Susceptibility Testing (EUCAST) recommendations, 2021 [43]. The disks impregnated with the following antimicrobial agents were tested: imipenem (10 μg), meropenem (10 µg), ciprofloxacin (5 µg), levofloxacin (5 µg), ceftazidime (10 µg), cefepime (30 µg), amikacin (30 µg), piperacillin/tazobactam (30/6 µg), aztreonam (30 µg), ticarcillin (75 µg), ceftazidime-avibactam (10–4 µg), and ceftolozane/tazobactam (38–10 µg) (BioRad, Watford, UK). Minimum inhibitory concentrations (MICs) for colistin, imipenem, and meropenem were evaluated by ComASP Colistin (Liofilchem, Roseto, Italy) and Gradient strip test (Liofilchem, Roseto, Italy), respectively. *P. aeruginosa* ATCC 27853 was used as the control strain in the antibiotic susceptibility testing. MDR *P. aeruginosa* were defined as isolates that tested resistant to at least one antimicrobial agent of three or more different classes. Extensively drug-resistant (XDR) *P. aeruginosa* were defined as a subset of MDR isolates that tested non-susceptible to at least one antimicrobial agent of five different classes. *P. aeruginosa* was defined as pandrug-resistant (PDR) when the organism was resistant to all tested agents.

### 4.3. PCR Amplification of Resistance Genes

All carbapenem-resistant *P. aeruginosa* isolates were screened by PCR following Sanger sequencing in case of positive PCR, for the presence of the three genes encoding MBL, *bla_VIM_*, *bla_IMP_*, and *bla_NDM_* [44]. Thereafter, all *P. aeruginosa* harboring MBL genes were subjected to the PCR for the detection of the genes encoding the most common ESBL-type enzymes in *P. aeruginosa*, namely *bla_CTX-M_*, *bla_PER_*, *bla_TEM_*, *bla_SHV_*, *bla_VEB_*, and *bla_GES_* [45,46,47,48]. The primer pairs used in the study are shown in Supplementary Table S1.

### 4.4. Multilocus Sequence Typing

The MLST analysis for *P. aeruginosa* harboring beta-lactamase genes was performed as described previously [49]. The primer pairs used for MLST analysis are shown in Supplementary Table S1. The obtained sequences of each housekeeping gene were compared with sequences in the MLST database (https://pubmlst.org/paeruginosa/, accessed on 1 July 2022) for the assignment of allelic numbers and STs.

A Pseudomonas Genome database search (Available at: www.pseudomonas.com, accessed on 1 July 2022 [50]) for *P. aeruginosa* isolates belonging to the same STs was carried out to create a database to use for phylogenetic analysis and to determine the clones’ geographical distribution. For these strains, information relative to the antibiotic resistance profile emphasizing the presence of carbapenems resistance genes was retrieved.

### 4.5. Phylogenomic and Genomic Analysis of bla_NDM_-Positive Strains

Four *bla_NDM_*-positive *P. aeruginosa* isolates were randomly selected for whole-genome sequencing (WGS) and further analysis (Illumina HiSeq paired-end and Oxford Nanopore MinION). De novo assemblies of the four *P. aeruginosa* genomes were generated using Unicycler v.0.4.8 [51]. The quality and completeness of the genome assemblies were assessed by testing the contamination with ContEst16S [52], and visualization with Bandage [53]. The genomes were then annotated using Prokka v1.12 [54], and rRNAs were identified by RNAmmer v1.2 [55]. PlasmidFinder 2.1, available on the Center for Genomic Epidemiology database was used to identify plasmids in whole genome sequencing data [56]. The core genes of the four *P. aeruginosa* genomes and of 161 (152 ST235 + nine ST654) previously published genomes of the same STs available in the NCBI Pathogen Detection database with a known geographical location were analyzed using Roary v3.13.0 [57], with a 95% minimum blastp identity and a 99% core definition threshold. Then, the SNPs of core genes were called to reduce the computational complexity for phylogenetic tree construction using SNP-sites v2.4.1 [58]. Afterward, a phylogenetic tree was built by raxmlHPC-PTHREADS v8.2.12 with the neighbor-joining method using 1000 bootstraps [59]. Visualization of the phylogenetic tree was performed using MEGA v.11 [60]. Genetic environment analysis of the flanking regions of the detected *bla_NDM_* genes was manually inspected using Geneious Prime software and ISfinder database [61].

### 4.6. Statistical Analysis

Statistical analysis was performed using SPSS version 14.0 (Chicago, IL, USA). The χ^2^ test and Fisher’s exact test were used to compare categorical data. A *p*  ≤  0.05 value indicated significantly different prevalence values.

## 5. Conclusions

In summary, this first Serbia nationwide study highlights the high frequency of several clones of MDR and XDR *P. aeruginosa*, the majority harboring β-lactamase encoding genes belonging to class B (MBL: *bla_NDM-1_*) and A (ESBL: *bla_PER-1,_ bla_GES-5_*) beta-lactamases. Based on our findings, we conclude that globally disseminated ST235 also predominated throughout the country. The obtained results go beyond previous reports, showing the emergence of *P. aeruginosa* ST654 in Serbia. The association of the major genetic lineages and multi-resistant phenotypes is a cause for considerable concern.

## Figures and Tables

**Table 1:** Demographic and clinical characteristics of *Pseudomonas aeruginosa*-infected study patients.

Characteristic	No. (%)
**Gender**	
Male gender	172 (53.8)
Female gender	148 (46.2)
**Admission ward**	
Intensive care unit	126 (39.4)
Thoracic surgery	3 (0.9)
Orthopedic surgery	3 (0.9)
Plastic surgery	6 (1.9)
Cardiovascular surgery	39 (12.2)
Neurosurgery	10 (3.1)
General surgery	29 (9.1)
Urology surgery	3 (0.9)
Internal wards	52 (16.3)
Department for COVID-19-positive patients	23 (7.2)
Geriatric department	19 (5.9)
Department of Oncology	7 (2.2)
**Type of specimen**	
Tracheal aspirate	74 (23.1)
Bronchoalveolar lavage	26 (8.1)
Sputum	20 (6.3)
Wound specimen	108 (33.8)
Blood	21 (6.6)
Urine	71 (22.2)
**Comorbidity**	
Malignancy	36 (11.2)
Chronic venous insufficiency	18 (5.6)
Heart insufficiency	18 (5.6)
Diabetes	23 (7.2)
COVID-19 pneumonia	23 (7.2)
**Invasive procedures**	
Any surgical procedure	93 (29.1)
Mechanical ventilation	72 (22.5)
Central venous catheter	42 (13.1)
Urinary catheter	139 (43.4)

## Data Availability

The genome sequences of four randomly selected *bla_NDM_*-positive *P. aeruginosa* (NDM1_1, NDM1_2, NDM1_3, and NDM1_4) isolates associated with this study have been submitted to the National Center for Biotechnology Information (NCBI) site, under BioProject Accession number PRJNA753000.

## Supplementary Materials

The following supporting information can be downloaded at: https://www.mdpi.com/article/10.3390/ijms24021519/s1.
