# Supplementary material for: Dissemination of Metallo-β-Lactamase-Producing Pseudomonas aeruginosa in Serbian Hospital Settings: Expansion of ST235 and ST654 Clones
Source: Int J Mol Sci. 2023 Jan 12;24(2):1519. doi: 10.3390/ijms24021519 (PMC9863560; doi:10.3390/ijms24021519)

Figure S1. Gel electrophoresis showing PCR amplification of beta-lactamases encoding genes

a) *bla*$_{NDM}$ gene

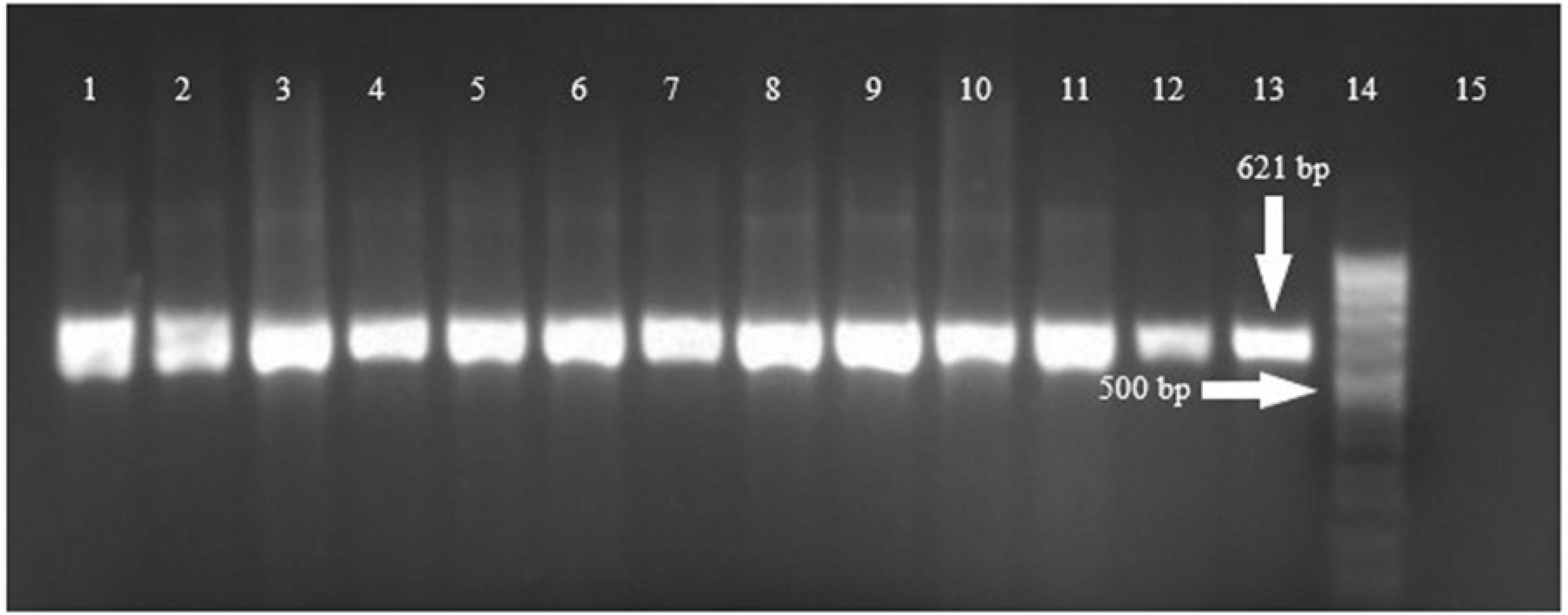

Lane 1-12: DNA samples
Lane 13: Positive control
Lane 14: DNA marker
Lane 15: Negative control

b) *bla*$_{PER}$ gene

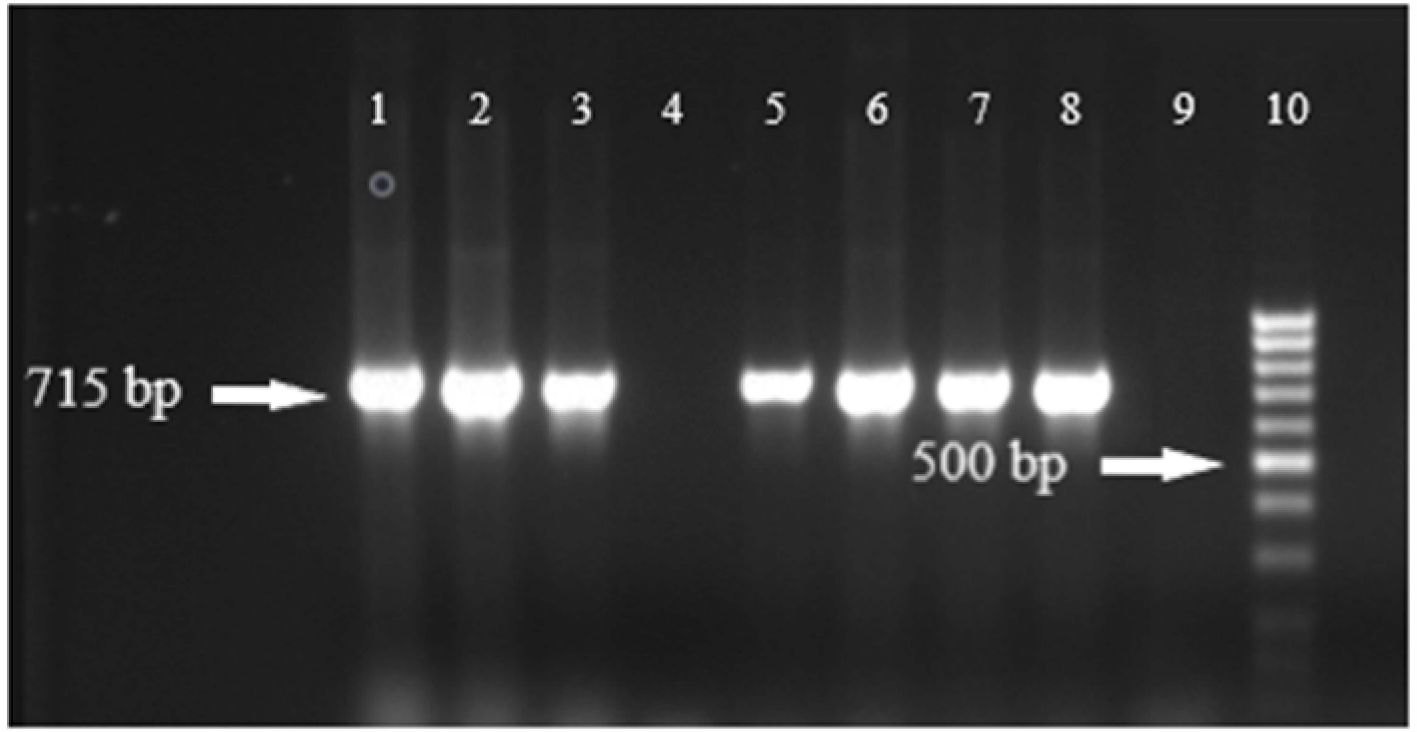

Lane 1-7: DNA samples
Lane 8: Positive control
Lane 9: Negative control
Lane 10: DNA marker

Figure S2. Geographical map showing the locations of the participating hospitals. Hospital locations are indicated by blue circles; hospitals where MBL-positive *Pseudomonas aeruginosa* were collected are identified by yellow asterisks

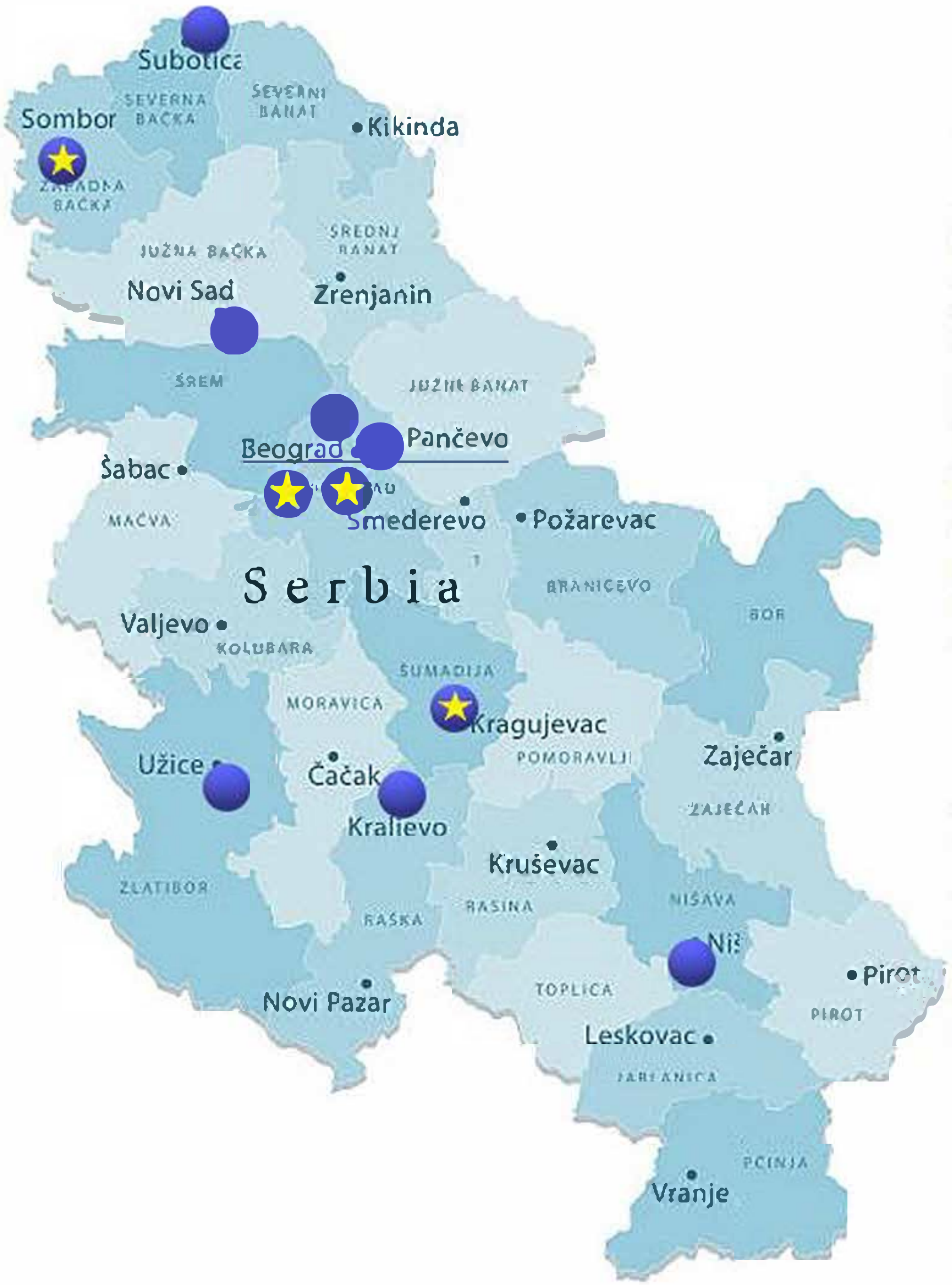

Clinical isolates of *Pseudomonas aeruginosa* (N=320)

Locations of 11 hospitals:
Belgrade (Clinical Center of Serbia, Medical Military Academy, University Hospital Center Dr Dragisa Misovic, University Hospital Medical Center Bezanijska kosa)
Novi Sad
Sombor
Subotica
Kragujevac
Kraljevo
Uzice
Nis

MBL-positive *Pseudomonas aeruginosa* (N=31)

Locations of hospitals:
Belgrade
Sombor
Kragujevac

Figure S3. Detected beta-lactamase resistance profiles of genomes affiliated with sequence types ST235 and ST654

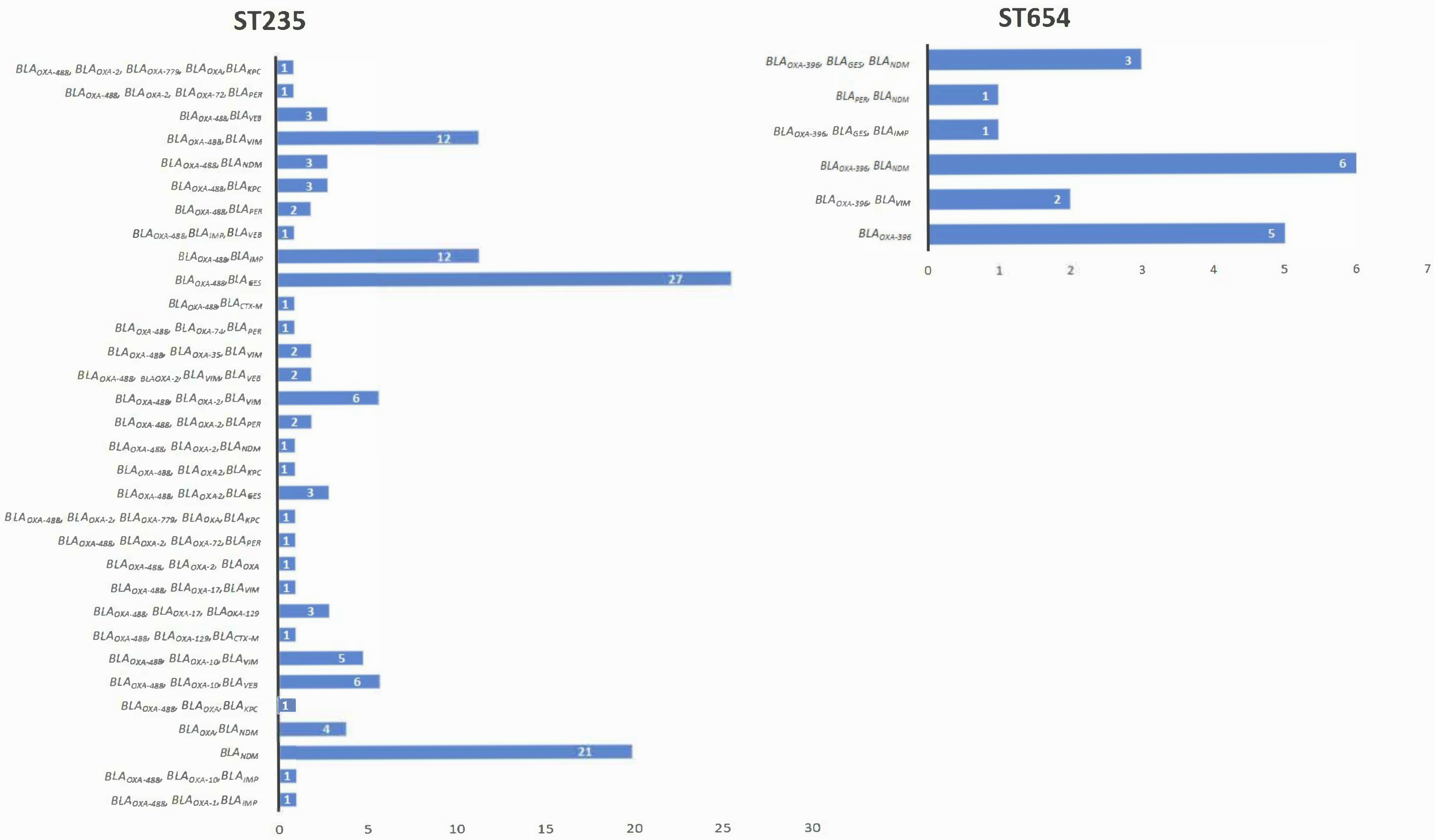

Supplement: Supplementary file 1 [file ijms-24-01519-s001.zip › Supplementary Figures S1, S2 and S3 revised.pdf]
