# Supplementary material for: Dissemination of Metallo-β-Lactamase-Producing Pseudomonas aeruginosa in Serbian Hospital Settings: Expansion of ST235 and ST654 Clones
Source: Int J Mol Sci. 2023 Jan 12;24(2):1519. doi: 10.3390/ijms24021519 (PMC9863560; doi:10.3390/ijms24021519)
Supplement: Supplementary file 1 [file ijms-24-01519-s001.zip › Supplementary Tables S1-S3.pdf]

Supplementary Table S1. Primers used in this study

**Primer**	**Sequence**	**Amplicon size (bp)**	**Reference**
NDM-F	5′-GGTTTGGCGATCTGGTTTTC-3’	621	[1]
NDM-R	5′-CGGAATGGCTCATCACGATC-3’		
PER-F	5′-CCTGACGATCTGGAACCTTT-3’	715	[2]
PER-R	5′-GCAACCTGCGCAATGATAGC-3’		
VIM-F	5′-GATGGTGTTTGGTCGCATA-3’	390	[1]
VIM-R	5′-CGAATGCGCAGCACCAG-3’		
IMP-F	5′-GGAATAGAGTGGCTTAAYTCTC-3’	232	[1]
IMP-R	5′-GGTTTAAYAAAACAACCACC-3’		
CTX-M-F	5′-AAAAATCACTGCGCCAGTTC-3’	415	[3]
CTX-M-R	5′-AGCTTATTCATCGCCACGTT-3’		
TEM-F	5′-ATGATGATTCAACATTTCCG-3’	1080	[4]
TEM-R	5′-CCAATGCTTAATCAGTGAGG-3’		
SHV-F	5′-CGCCGGGTTATTCTTATTTG-3’	1069	[5]
SHV-R	5′-CCACGTTTATGGCGTTACCT-3’		
GES-F	5′-ATGCGCTTCATTCACGCAC-3’	840	[2]
GES-R	5′-AACTCATCCTGAGCACGGAC-3’		
VEB-F	5′-ATTTCCCGATGCAAAGCGT-3’	360	[2]
VEB-R	5′-CCAACAGCGATGAACAAACT-3’		
acsA-F	5′-ACCTGGTGTACGCCTCGCTGAC-3’	842	[6]
acsA-R	5′-GACATAGATGCCCTGCCCCTTGAT-3’		
aroE-F	5′-TGGGGCTATGACTGGAAACC-3’	825	[6]
aroE-R	5′-TAACCCGGTTTTGTGATTCCTACA-3’		
guaA-F	5′-CGGCCTCGACGTGTGGATGA-3’	940	[6]
guaA-R	5′-GAACGCCTGGCTGGTCTTGTGGTA-3’		
mutL-F	5′-CCAGATCGCCGCCGGTGAGGTG-3’	940	[6]
mutL-R	5′-CAGGGTGCCATAGAGGAAGTC-3’		
nuoD-F	5′-ACCGCCACCCGTACTG-3’	1042	[6]
nuoD-R	5′-TCTCGCCCATCTTGACCA-3’		
ppsA-F	5′-GGTCGCTCGGTCAAGGTAGTGG-3’	989	[6]
ppsA-R	5′-GGGTTCTCTTCTTCCGGCTCGTAG-3’		
trpE-F	5′-GCGGCCCAGGGTCGTGAG-3’	811	[6]
trpE-R	5′-CCCGGCGCTTGTTGATGGTT-3’		
acsA-F IS	5′-GCCACACCTACATCGTCTAT-3’	390	[6]
acsA-R IS	5′-AGGTTGCCGAGGTTGTCCAC-3’		
aroE-F IS	5′-ATGTCACCGTGCCGTTCAAG-3’	495	[6]
aroE-R IS	5′-TGAAGGCAGTCGGTTCCTTG-3’		
guaA-F IS	5′-AGGTCGGTTCCTCCAAGGTC-3’	372	[6]
guaA-R IS	5′-GACGTTGTGGTGCGACTTGA-3’		
mutL-F IS	5′-AGAAGACCGAGTTCGACCAT-3’	441	[6]
mutL-R IS	5′-GGTGCCATAGAGGAAGTCAT-3’		
nuoD-F IS	5′-ACGGCGAGAACGAGGACTAC-3’	366	[6]
nuoD-R IS	5′-TGGCGGTCGGTGAAGGTGAA-3’		
ppsA-F IS	5′-GGTGACGACGGCAAGCTGTA-3’	369	[6]
ppsA-R IS	5′-GTATCGCCTTCGGCACAGGA-3’		
trpE-F IS	5′-TTCAACTTCGGCGACTTCCA-3’	441	[6]
trpE-R IS	5′-GGTGTCCATGTTGCCGTTCC-3’		

1. Poirel L, Walsh TR, Cuvillier V, Nordmann P. Multiplex PCR for detection of acquired carbapenemase genes. DiagnMicrobiol Infect Dis. 2011;70(1):119-23.
2. Opazo A, Sonnevend A, Lopes B, Hamouda A, Ghazawi A, Pal T et al. Plasmid-encoded PER-7 -lactamase responsible for ceftazidime resistance in *Acinetobacter baumannii* isolated in the United Arab Emirates. J AntimicrobChemother. 2012;67(7):1619-1622.
3. Celenza G, Pellegrini C, Caccamo M, Segatore B, Amicosante G, Perilli M. Spread of bla(CTX-M-type) and bla(PER-2) beta-lactamase genes in clinical isolates from Bolivian hospitals. J Antimicrob Chemother. 2006 May;57(5):975-8.
4. Yoshizumi A, Ishii Y, Aoki K, Testa R, Nichols WW, Tateda K. In vitro susceptibility of characterized β-lactamase-producing Gram-negative bacteria isolated in Japan to ceftazidime-, ceftaroline-, and aztreonam-avibactam combinations. J Infect Chemother. 2015;21(2):148-51.
5. Bae I, Jang S, Kim J, Jeong S, Cho B, Lee K. Interspecies dissemination of the bla gene encoding PER-1 extended-spectrum β-lactamase. Antimicrob Agents Chemother. 2011;55(3):1305-1307.
6. Curran B, Jonas D, Grundmann H, Pitt T, Dowson CG. Development of a multilocus sequence typing scheme for the opportunistic pathogen *Pseudomonas aeruginosa*. J Clin Microbiol.2004;42:5644e9

Supplementary Table S2. Genome assembly quality of *bla*$_{NDM}$-positive *Pseudomonas. aeruginosa* isolates

Isolate	NDM-1	NDM-2	NDM-3	NDM-4
ST	ST654	ST235	ST235	ST235
Number of contigs	35	1	2	2
Genome size (Mbp)	6.9	7.0	7.1	7.1
Coverage	300x	100x	100x	100x
Contig N50 (bp)	3615812	7038982	7124494	7093670
Contamination level values	0.11	0.43	0.59	0.59
Accession number	GCF_020404785.1	GCF_022559565.1	GCF_020404825.1	GCF_020404715.1

ST – sequence type

Supplementary Table S3. ***Pseudomonas aeruginosa*** **genomes used in the phylogenomic analysis**

Strain label on tree	Assembly	Location	Sequence Type
GCA_000794805.1	https://www.ncbi.nlm.nih.gov/assembly/GCA_000794805.1/	Philippines	235
GCA_000795805.1	https://www.ncbi.nlm.nih.gov/assembly/GCA_000795805.1/	Greece	235
GCA_001086635.1	https://www.ncbi.nlm.nih.gov/assembly/GCA_001086635.1/	USA	235
GCA_002188505.1	https://www.ncbi.nlm.nih.gov/assembly/GCA_002188505.1/	Portugal	235
GCF_000786485.1	https://www.ncbi.nlm.nih.gov/assembly/GCF_000786485.1/	Romania	235
GCF_000790725.1	https://www.ncbi.nlm.nih.gov/assembly/GCF_000790725.1/	Croatia	235
GCF_000791915.1	https://www.ncbi.nlm.nih.gov/assembly/GCF_000791915.1/	Croatia	235
GCF_000794055.1	https://www.ncbi.nlm.nih.gov/assembly/GCF_000794055.1/	Croatia	235
GCF_000794185.1	https://www.ncbi.nlm.nih.gov/assembly/GCF_000794185.1/	Argentina	235
GCF_000794545.1	https://www.ncbi.nlm.nih.gov/assembly/GCF_000794545.1/	Mexico	235
GCF_000794555.1	https://www.ncbi.nlm.nih.gov/assembly/GCF_000794555.1/	Romania	235
GCF_000794645.1	https://www.ncbi.nlm.nih.gov/assembly/GCF_000794645.1/	Romania	235
GCF_000794785.1	https://www.ncbi.nlm.nih.gov/assembly/GCF_000794785.1/	Philippines	235
GCF_023904315.1	https://www.ncbi.nlm.nih.gov/assembly/GCF_023904315.1/	Philippines	235
GCF_000794865.1	https://www.ncbi.nlm.nih.gov/assembly/GCF_000794865.1/	USA	235
GCF_000794945.1	https://www.ncbi.nlm.nih.gov/assembly/GCF_000794945.1/	Italy	235
GCF_000794985.1	https://www.ncbi.nlm.nih.gov/assembly/GCF_000794985.1/	USA	235
GCF_000795435.1	https://www.ncbi.nlm.nih.gov/assembly/GCF_000795435.1/	India	235
GCF_000795605.1	https://www.ncbi.nlm.nih.gov/assembly/GCF_000795605.1/	USA	235
GCF_000795685.1	https://www.ncbi.nlm.nih.gov/assembly/GCF_000795685.1/	Argentina	235
GCF_000795705.1	https://www.ncbi.nlm.nih.gov/assembly/GCF_000795705.1/	Italy	235
GCF_000795725.1	https://www.ncbi.nlm.nih.gov/assembly/GCF_000795725.1/	Mexico	235
GCF_000795765.1	https://www.ncbi.nlm.nih.gov/assembly/GCF_000795765.1/	France	235
GCF_000795805.1	https://www.ncbi.nlm.nih.gov/assembly/GCF_000795805.1/	Greece	235
GCF_000795815.1	https://www.ncbi.nlm.nih.gov/assembly/GCF_000795815.1/	Greece	235
GCF_000795955.1	https://www.ncbi.nlm.nih.gov/assembly/GCF_000795955.1/	Colombia	235
GCF_000796165.1	https://www.ncbi.nlm.nih.gov/assembly/GCF_000796165.1/	Greece	235
GCF_000796225.1	https://www.ncbi.nlm.nih.gov/assembly/GCF_000796225.1/	USA	235
GCF_000796245.1	https://www.ncbi.nlm.nih.gov/assembly/GCF_000796245.1/	Colombia	235
GCF_000797025.1	https://www.ncbi.nlm.nih.gov/assembly/GCF_000797025.1/	Colombia	235
GCF_000829255.1	https://www.ncbi.nlm.nih.gov/assembly/GCF_000829255.1/	Japan	235
GCF_000829275.1	https://www.ncbi.nlm.nih.gov/assembly/GCF_000829275.1/	Japan	235
GCF_001086645.1	https://www.ncbi.nlm.nih.gov/assembly/GCF_001086645.1/	USA	235
GCF_001086655.1	https://www.ncbi.nlm.nih.gov/assembly/GCF_001086655.1/	USA	235
GCF_001086735.1	https://www.ncbi.nlm.nih.gov/assembly/GCF_001086735.1/	USA	235
GCF_001086745.1	https://www.ncbi.nlm.nih.gov/assembly/GCF_001086745.1/	USA	235
GCF_001086805.1	https://www.ncbi.nlm.nih.gov/assembly/GCF_001086805.1/	USA	235
GCF_001449645.1	https://www.ncbi.nlm.nih.gov/assembly/GCF_001449645.1/	France	235

GCF_001449765.1	https://www.ncbi.nlm.nih.gov/assembly/GCF_001449765.1/	France	235
GCF_001450265.1	https://www.ncbi.nlm.nih.gov/assembly/GCF_001450265.1/	France	235
GCF_001450505.1	https://www.ncbi.nlm.nih.gov/assembly/GCF_001450505.1/	France	235
GCF_001450545.1	https://www.ncbi.nlm.nih.gov/assembly/GCF_001450545.1/	France	235
GCF_001450995.1	https://www.ncbi.nlm.nih.gov/assembly/GCF_001450995.1/	France	235
GCF_001451125.1	https://www.ncbi.nlm.nih.gov/assembly/GCF_001451125.1/	France	235
GCF_001451485.1	https://www.ncbi.nlm.nih.gov/assembly/GCF_001451485.1/	USA	235
GCF_001451785.1	https://www.ncbi.nlm.nih.gov/assembly/GCF_001451785.1/	USA	235
GCF_001451815.1	https://www.ncbi.nlm.nih.gov/assembly/GCF_001451815.1/	USA	235
GCF_001451885.1	https://www.ncbi.nlm.nih.gov/assembly/GCF_001451885.1/	USA	235
GCF_001451985.1	https://www.ncbi.nlm.nih.gov/assembly/GCF_001451985.1/	USA	235
GCF_001452025.1	https://www.ncbi.nlm.nih.gov/assembly/GCF_001452025.1/	USA	235
GCF_001452045.1	https://www.ncbi.nlm.nih.gov/assembly/GCF_001452045.1/	USA	235
GCF_001452055.1	https://www.ncbi.nlm.nih.gov/assembly/GCF_001452055.1/	USA	235
GCF_001452285.1	https://www.ncbi.nlm.nih.gov/assembly/GCF_001452285.1/	USA	235
GCF_001452305.1	https://www.ncbi.nlm.nih.gov/assembly/GCF_001452305.1/	USA	235
GCF_001452575.1	https://www.ncbi.nlm.nih.gov/assembly/GCF_001452575.1/	USA	235
GCF_001452675.1	https://www.ncbi.nlm.nih.gov/assembly/GCF_001452675.1/	USA	235
GCF_001452805.1	https://www.ncbi.nlm.nih.gov/assembly/GCF_001452805.1/	Germany	235
GCF_001453345.1	https://www.ncbi.nlm.nih.gov/assembly/GCF_001453345.1/	USA	235
GCF_001453565.1	https://www.ncbi.nlm.nih.gov/assembly/GCF_001453565.1/	USA	235
GCF_001453595.1	https://www.ncbi.nlm.nih.gov/assembly/GCF_001453595.1/	USA	235
GCF_001454045.1	https://www.ncbi.nlm.nih.gov/assembly/GCF_001454045.1/	Turkey	235
GCF_001454305.1	https://www.ncbi.nlm.nih.gov/assembly/GCF_001454305.1/	Bulgaria	235
GCF_001454595.1	https://www.ncbi.nlm.nih.gov/assembly/GCF_001454595.1/	Hungary	235
GCF_001623945.1	https://www.ncbi.nlm.nih.gov/assembly/GCF_001623945.1/	Australia	235
GCF_001623955.1	https://www.ncbi.nlm.nih.gov/assembly/GCF_001623955.1/	Australia	235
GCF_001623985.1	https://www.ncbi.nlm.nih.gov/assembly/GCF_001623985.1/	Australia	235
GCF_001680745.1	https://www.ncbi.nlm.nih.gov/assembly/GCF_001680745.1/	Brazil	235
GCF_001756435.1	https://www.ncbi.nlm.nih.gov/assembly/GCF_001756435.1/	Belgium	235
GCF_001920965.1	https://www.ncbi.nlm.nih.gov/assembly/GCF_001920965.1/	France	235
GCF_001921005.1	https://www.ncbi.nlm.nih.gov/assembly/GCF_001921005.1/	Argentina	235
GCF_001921015.1	https://www.ncbi.nlm.nih.gov/assembly/GCF_001921015.1/	Argentina	235
GCF_001921025.1	https://www.ncbi.nlm.nih.gov/assembly/GCF_001921025.1/	South Africa	235
GCF_001921065.1	https://www.ncbi.nlm.nih.gov/assembly/GCF_001921065.1/	USA	235
GCF_001921085.1	https://www.ncbi.nlm.nih.gov/assembly/GCF_001921085.1/	Russia	235
GCF_001921095.1	https://www.ncbi.nlm.nih.gov/assembly/GCF_001921095.1/	Thailand	235
GCF_001921105.1	https://www.ncbi.nlm.nih.gov/assembly/GCF_001921105.1/	Russia	235
GCF_001921125.1	https://www.ncbi.nlm.nih.gov/assembly/GCF_001921125.1/	Russia	235
GCF_001921165.1	https://www.ncbi.nlm.nih.gov/assembly/GCF_001921165.1/	Russia	235
GCF_001921175.1	https://www.ncbi.nlm.nih.gov/assembly/GCF_001921175.1/	Nigeria	235
GCF_002003595.1	https://www.ncbi.nlm.nih.gov/assembly/GCF_002003595.1/	Sudan	235
GCF_002193975.1	https://www.ncbi.nlm.nih.gov/assembly/GCF_002193975.1/	USA	235
GCF_002283315.1	https://www.ncbi.nlm.nih.gov/assembly/GCF_002283315.1/	Brazil	235
GCF_002406265.1	https://www.ncbi.nlm.nih.gov/assembly/GCF_002406265.1/	Australia	235

GCF_002406305.1	https://www.ncbi.nlm.nih.gov/assembly/GCF_002406305.1/	Australia	235
GCF_002406335.1	https://www.ncbi.nlm.nih.gov/assembly/GCF_002406335.1/	Australia	235
GCF_002406345.1	https://www.ncbi.nlm.nih.gov/assembly/GCF_002406345.1/	Australia	235
GCF_002812905.1	https://www.ncbi.nlm.nih.gov/assembly/GCF_002812905.1/	USA	235
GCF_002812925.1	https://www.ncbi.nlm.nih.gov/assembly/GCF_002812925.1/	USA	235
GCF_003325585.1	https://www.ncbi.nlm.nih.gov/assembly/GCF_003325585.1/	Brazil	235
GCF_003397565.1	https://www.ncbi.nlm.nih.gov/assembly/GCF_003397565.1/	Lebanon	235
GCF_003410555.1	https://www.ncbi.nlm.nih.gov/assembly/GCF_003410555.1/	USA	235
GCF_003411005.1	https://www.ncbi.nlm.nih.gov/assembly/GCF_003411005.1/	USA	235
GCF_003411565.1	https://www.ncbi.nlm.nih.gov/assembly/GCF_003411565.1/	USA	235
GCF_003411635.1	https://www.ncbi.nlm.nih.gov/assembly/GCF_003411635.1/	USA	235
GCF_003412035.1	https://www.ncbi.nlm.nih.gov/assembly/GCF_003412035.1/	USA	235
GCF_003433235.1	https://www.ncbi.nlm.nih.gov/assembly/GCF_003433235.1/	Colombia	235
GCF_003585105.1	https://www.ncbi.nlm.nih.gov/assembly/GCF_003585105.1/	Hong Kong	235
GCF_003632055.1	https://www.ncbi.nlm.nih.gov/assembly/GCF_003632055.1/	Estonia	235
GCF_003641125.1	https://www.ncbi.nlm.nih.gov/assembly/GCF_003641125.1/	USA	235
GCF_003833565.1	https://www.ncbi.nlm.nih.gov/assembly/GCF_003833565.1/	United Kingdom	235
GCF_003834495.1	https://www.ncbi.nlm.nih.gov/assembly/GCF_003834495.1/	USA	235
GCF_003834545.1	https://www.ncbi.nlm.nih.gov/assembly/GCF_003834545.1/	USA	235
GCF_003835385.1	https://www.ncbi.nlm.nih.gov/assembly/GCF_003835385.1/	Thailand	235
GCF_003837675.1	https://www.ncbi.nlm.nih.gov/assembly/GCF_003837675.1/	Georgia	235
GCF_003934215.1	https://www.ncbi.nlm.nih.gov/assembly/GCF_003934215.1/	Thailand	235
GCF_003934225.1	https://www.ncbi.nlm.nih.gov/assembly/GCF_003934225.1/	Thailand	235
GCF_003936835.1	https://www.ncbi.nlm.nih.gov/assembly/GCF_003936835.1/	Pakistan	235
GCF_003936865.1	https://www.ncbi.nlm.nih.gov/assembly/GCF_003936865.1/	Pakistan	235
GCF_003936975.1	https://www.ncbi.nlm.nih.gov/assembly/GCF_003936975.1/	Pakistan	235
GCF_003940575.1	https://www.ncbi.nlm.nih.gov/assembly/GCF_003940575.1/	Pakistan	235
GCF_003940585.1	https://www.ncbi.nlm.nih.gov/assembly/GCF_003940585.1/	Pakistan	235
GCF_003940675.1	https://www.ncbi.nlm.nih.gov/assembly/GCF_003940675.1/	Pakistan	235
GCF_003968165.1	https://www.ncbi.nlm.nih.gov/assembly/GCF_003968165.1/	USA	235
GCF_003968225.1	https://www.ncbi.nlm.nih.gov/assembly/GCF_003968225.1/	USA	235
GCF_003968235.1	https://www.ncbi.nlm.nih.gov/assembly/GCF_003968235.1/	USA	235
GCF_003968295.1	https://www.ncbi.nlm.nih.gov/assembly/GCF_003968295.1/	USA	235
GCF_003968305.1	https://www.ncbi.nlm.nih.gov/assembly/GCF_003968305.1/	USA	235
GCF_003969485.1	https://www.ncbi.nlm.nih.gov/assembly/GCF_003969485.1/	USA	235
GCF_003977535.1	https://www.ncbi.nlm.nih.gov/assembly/GCF_003977535.1/	Egypt	235
GCF_004349915.1	https://www.ncbi.nlm.nih.gov/assembly/GCF_004349915.1/	Italy	235
GCF_004349975.1	https://www.ncbi.nlm.nih.gov/assembly/GCF_004349975.1/	Italy	235
GCF_004350095.1	https://www.ncbi.nlm.nih.gov/assembly/GCF_004350095.1/	Italy	235
GCF_004350115.1	https://www.ncbi.nlm.nih.gov/assembly/GCF_004350115.1/	Italy	235
GCF_004350125.1	https://www.ncbi.nlm.nih.gov/assembly/GCF_004350125.1/	Italy	235
GCF_004350135.1	https://www.ncbi.nlm.nih.gov/assembly/GCF_004350135.1/	Italy	235
GCF_004350275.1	https://www.ncbi.nlm.nih.gov/assembly/GCF_004350275.1/	Italy	235
GCF_004350355.1	https://www.ncbi.nlm.nih.gov/assembly/GCF_004350355.1/	Italy	235
GCF_004350485.1	https://www.ncbi.nlm.nih.gov/assembly/GCF_004350485.1/	Italy	235

GCF_004350565.1	https://www.ncbi.nlm.nih.gov/assembly/GCF_004350565.1/	Italy	235
GCF_004350925.1	https://www.ncbi.nlm.nih.gov/assembly/GCF_004350925.1/	Italy	235
GCF_004350945.1	https://www.ncbi.nlm.nih.gov/assembly/GCF_004350945.1/	Italy	235
GCF_004350955.1	https://www.ncbi.nlm.nih.gov/assembly/GCF_004350955.1/	Italy	235
GCF_004351125.1	https://www.ncbi.nlm.nih.gov/assembly/GCF_004351125.1/	Italy	235
GCF_004351205.1	https://www.ncbi.nlm.nih.gov/assembly/GCF_004351205.1/	Italy	235
GCF_004351415.1	https://www.ncbi.nlm.nih.gov/assembly/GCF_004351415.1/	Italy	235
GCF_004351435.1	https://www.ncbi.nlm.nih.gov/assembly/GCF_004351435.1/	Italy	235
GCF_004351685.1	https://www.ncbi.nlm.nih.gov/assembly/GCF_004351685.1/	Italy	235
GCF_004351755.1	https://www.ncbi.nlm.nih.gov/assembly/GCF_004351755.1/	Italy	235
GCF_004354095.1	https://www.ncbi.nlm.nih.gov/assembly/GCF_004354095.1/	Italy	235
GCF_005048525.1	https://www.ncbi.nlm.nih.gov/assembly/GCF_005048525.1/	USA	235
GCF_007559125.1	https://www.ncbi.nlm.nih.gov/assembly/GCF_007559125.1/	China	235
GCF_007954175.1	https://www.ncbi.nlm.nih.gov/assembly/GCF_007954175.1/	USA	235
GCF_008195485.1	https://www.ncbi.nlm.nih.gov/assembly/GCF_008195485.1/	Sweden	235
GCF_008244545.1	https://www.ncbi.nlm.nih.gov/assembly/GCF_008244545.1/	France	235
GCF_008244625.1	https://www.ncbi.nlm.nih.gov/assembly/GCF_008244625.1/	Sweden	235
GCF_008244635.1	https://www.ncbi.nlm.nih.gov/assembly/GCF_008244635.1/	Sweden	235
GCF_008330985.1	https://www.ncbi.nlm.nih.gov/assembly/GCF_008330985.1/	Russia	235
GCF_008370085.1	https://www.ncbi.nlm.nih.gov/assembly/GCF_008370085.1/	Russia	235
GCF_009299585.1	https://www.ncbi.nlm.nih.gov/assembly/GCF_009299585.1/	Lebanon	235
GCF_009299655.1	https://www.ncbi.nlm.nih.gov/assembly/GCF_009299655.1/	Lebanon	235
GCF_009788935.1	https://www.ncbi.nlm.nih.gov/assembly/GCF_009788935.1/	Saudi Arabia	235
GCF_009788955.1	https://www.ncbi.nlm.nih.gov/assembly/GCF_009788955.1/	Saudi Arabia	235
NDM1_2	https://www.ncbi.nlm.nih.gov/assembly/GCF_022559565.1/	Serbia	235
NDM1_3	https://www.ncbi.nlm.nih.gov/assembly/GCF_020404825.1/	Serbia	235
NDM1_4	https://www.ncbi.nlm.nih.gov/assembly/GCF_020404715.1/	Serbia	235
GCA_013413775.1	https://www.ncbi.nlm.nih.gov/assembly/GCA_013413775.1/	Bulgaria	654
GCA_013413795.1	https://www.ncbi.nlm.nih.gov/assembly/GCA_013413795.1/	Bulgaria	654
GCA_014489175.1	https://www.ncbi.nlm.nih.gov/assembly/GCA_014489175.1/	Chile	654
GCF_000796405.1	https://www.ncbi.nlm.nih.gov/assembly/GCF_000796405.1/	France	654
GCF_001086755.1	https://www.ncbi.nlm.nih.gov/assembly/GCF_001086755.1/	USA	654
GCF_001482875.1	https://www.ncbi.nlm.nih.gov/assembly/GCF_001482875.1/	India	654
GCF_003571505.1	https://www.ncbi.nlm.nih.gov/assembly/GCF_003571505.1/	Canada	654
GCF_003969055.1	https://www.ncbi.nlm.nih.gov/assembly/GCF_003969055.1/	USA	654
GCF_009299705.1	https://www.ncbi.nlm.nih.gov/assembly/GCF_009299705.1/	Lebanon	654
NDM1_1	https://www.ncbi.nlm.nih.gov/assembly/GCF_020404785.1/	Serbia	654
